# Standardization of the protocol for oral cavity examination and collecting of the biological samples for microbiome research using the next-generation sequencing (NGS): own experience with the COVID-19 patients

**DOI:** 10.1038/s41598-024-53992-3

**Published:** 2024-02-14

**Authors:** Barbara Brzychczy-Sroka, Katarzyna Talaga-Ćwiertnia, Agnieszka Sroka-Oleksiak, Artur Gurgul, Elżbieta Zarzecka-Francica, Wojciech Ostrowski, Janusz Kąkol, Kamil Drożdż, Monika Brzychczy-Włoch, Joanna Zarzecka

**Affiliations:** 1 Department of Conservative Dentistry with Endodontics, Institute of Dentistry, Jagiellonian University Medical College, Kraków, Poland; 2 Chair of Microbiology, Department of Molecular Medical Microbiology, Faculty of Medicine, Jagiellonian University Medical College, Czysta 18, 31-121 Kraków, Poland; 3 Center for Experimental and Innovative Medicine, The University of Agriculture in Kraków, Kraków, Poland; 4 Department of Prosthodontics and Orthodontics, Institute of Dentistry, Jagiellonian University Medical College, Kraków, Poland; 5 University Hospital in Cracow, Temporary COVID Ward No. 1, Kraków, Poland

**Keywords:** Standardization, Calibration, Dentistry, Microbiota, Oral health, Oral hygiene, Next generation sequencing, Microbiology, Diseases

## Abstract

To date, publications have shown that compositions of oral microbiota differ depending on their habitats (e.g. tongue, tonsils, pharynx). The absence of set standards for the choice of the areas and conditions of material collection makes the oral microbiome one of the most difficult environments for a comparative analysis with other researchers, which is a meaningful limitation during an assessment of the potential effects of microorganisms as biomarkers in the courses of various human diseases. Therefore, standardisation of basic conditions of a dental examination and collection of material for the next generation sequencing (NGS) is worth attempting. The standardisation of the dental exam and collection of the clinical materials: saliva, swab from the tongue ridge, hard palate, palatine tonsils and oropharynx, supragingival plaque and subgingival plaque. Protocol involved the patients (n = 60), assigned to 3 groups: I—COVID-19 convalescents who received antibiotics, n = 17, II—COVID-19 convalescents, n = 23 and III—healthy individuals, n = 20. The collected biological samples were used to conduct NGS (16S rRNA). The conditions of patient preparation for collecting biological materials as well as the schedule of dental examination, were proposed. Based on the research conducted, we have indicated the dental indicators that best differentiate the group of COVID-19 patients (groups I and II) from healthy people (group III). These include the DMFT, D and BOP indices. The use of alpha and beta diversity analysis provided an overall insight into the diversity of microbial communities between specific niches and patient groups. The most different diversity between the studied group of patients (group II) and healthy people (group III) was noted in relation to the supragingival plaque. The order of activities during the dental exam as well as while collecting and securing clinical materials is particularly important to avoid technical errors and material contamination which may result in erroneous conclusions from the analyses of the results of sensitive tests such as the NGS. It has been shown that the dental indices: DMFT, D number, PI and BOP are the best prognostic parameters to assess the oral health. Based on beta diversity the most sensitive niche and susceptible to changes in the composition of the microbiota is the supragingival plaque. The procedures developed by our team can be applied as ready-to-use forms in studies conducted by other researchers.

## Introduction

A human oral cavity contains many different habitats such as the tongue, cheeks, soft and hard palates, tonsils, teeth and periodontal pockets^1,2^. Each of these oral regions is colonised by various microbial species: bacteria, fungi, viruses and archaea^1,3,4^. They create a complex ecosystem which affects the health and homeostasis maintenance not only in the oral cavity but in the whole body^5^. Development of a proper procedure for clinical sample collecting and analysing is necessary to determine microbial compositions in specific habitats and to assess their potential impact on the health and/or diseases. To date, oral microbial samples intended for metagenomic sequencing have been collected according to various protocols^1,6^.

The microbiota analysis with the use of the next generation sequencing (NGS) method allows for determination of the microbial qualitative composition and the share of microorganisms along with their assignment to the specific taxonomy levels. Compared to traditional microbiological methods utilising cultures in artificial media, the advantage of the NGS technology is its potential to detect small numbers of microorganisms which are extremely difficult to culture or culturing them in microbiological media is not possible^7^. In addition, the NGS ensures detection of dead microorganisms when their nucleic acids remain in a sample, which overcomes low sensitivity in culture-based conventional methods^8^ but is a limitation of this technique. Nevertheless, application of relevant parameters during the bioinformatic analysis is necessary to cut off possible readings which are traces of e.g. previous infections. To date, the NGS technology has been successfully used in dentistry to assess oral microbiome/microbiota in many diseases such as the periodontal disease^9^, oral squamous cell carcinoma^10^, lichen planus^11^, Behçet’s disease^12^, amelogenesis imperfecta^13^, epidermolysis bullosa^14^ and acrodermatitis enteropathica^14^. In this paper, the protocols of oral cavity examination and collection of selected oral samples (saliva, tongue swab, supra- and subgingival plaques, hard palate swab, palatine tonsil swab and posterior pharynx swab) for microbiome testing with the use of the 16S rRNA (V3–V4 regions) next generation sequencing method in the group of patients with COVID-19, developed by the authors of the paper, are presented.

## Materials and methods

### Patient preparation

**Figure 1:**
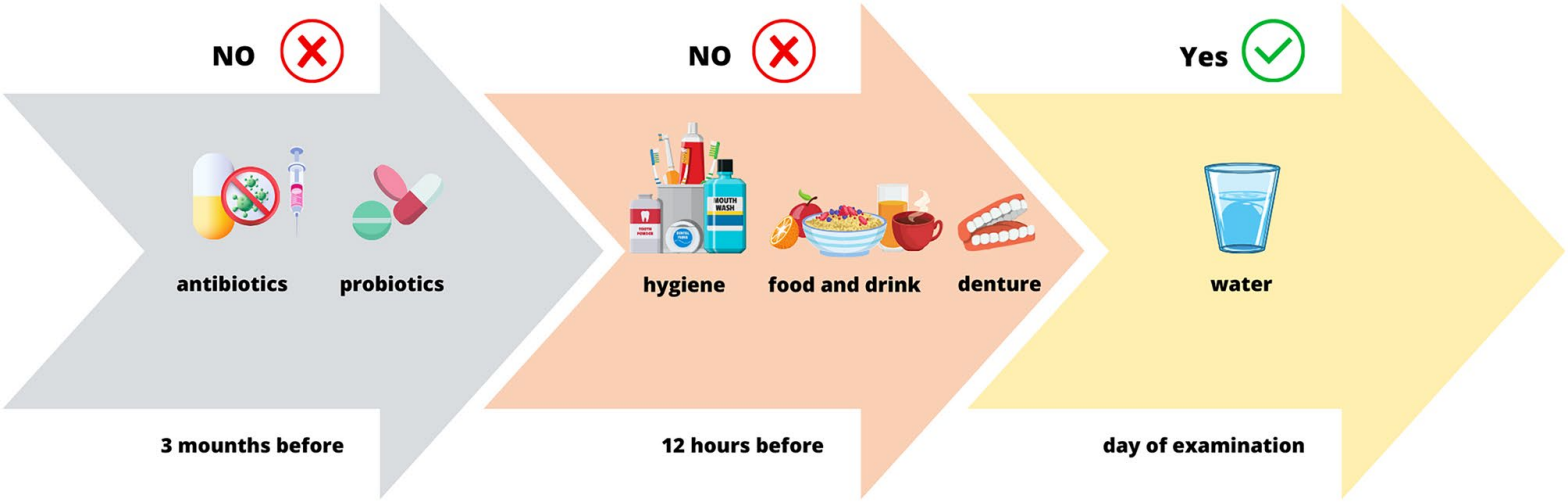
Preparing the patient for a dental examination and collecting materials for metagenomic testing.

Before a patient’s eligibility is confirmed and samples are collected for oral microbiome testing, the inclusion and exclusion criteria should be specified, considering e.g. the factors presented in Fig. 1. In order to ensure reliable results, it is important for the patient to be in the fasting state on the day of the oral examination and not to perform any oral hygiene procedures (Fig. 1). The patients who have mobile dentures should not use them overnight before the exam. Water consumption before the exam is acceptable as it facilitates collection of saliva from the patients, particularly from the elder who have problems with saliva secretion.

### Dental examination

**Figure 2:**
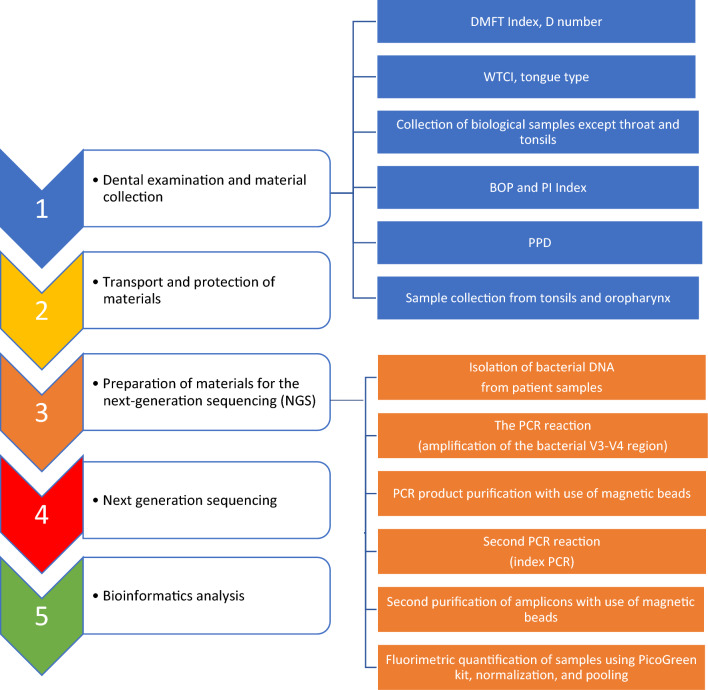
Steps during designing a clinical study for collecting the clinical materials for oral microbiome analysis.

The oral cavity examination and biological samples collection should be performed by a team of dentists who have been trained according to the designed protocol (Fig. 2, item 1). It would be best that all examinations were performed by one and the same dentist, or at least a team of calibrated dentists. Kappa values should ranged from 0.80 to 0.90^15^.

It is necessary to maintain the set order of activities and to use proposed by our team the Oral Cavity Examination Card systematizing it, providing information about the patient's overall health and oral health (Supp. 1). The dental examination begins with determination of the Decayed, Missing and Filled Teeth (DMFT) Index and the number of tooth lesions D (Decayed) (definitions and the methods of index calculations are explained in the Oral Cavity Examination Card and below). Next, we assess the Winkel Tongue Coating Index (WTCI) and the tongue type. The next step is collection of biological samples excluding the area of pharynx and tonsils. Then, we calculate the the Plaque index simplified (PI) and the Bleeding on Probing (BOP) Index and Periodontal Probing Depth (PPD) values. Finally, the samples from the tonsils and oropharynx are collected.

**Figure 3:**
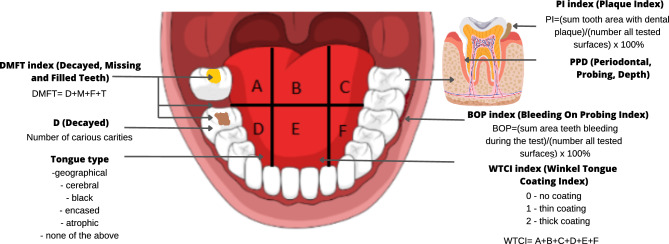
Dental indices evaluated during the dental investigation and collection of clinical materials for microbiome research.

During the dental exam, all dental indices which will characterise the study population and will help assess the oral hygiene and plaque-induced diseases (bacterial dental/gingival plaque) are worth assessment. To do this, the following values should be determined: the WTCI, tongue type, DMFT Index, D number, PI, BOP Index, PPD (Fig. 3).

The WTCI value should be calculated as follows: divide the tongue into six areas, determine the coating state (0—no coating, 1—thin coating, 2—thick coating) for each and sum up. The index range falls between 0 and 12. Next, the tongue type (geographical, cerebral, black, encased, atrophic, none of the listed) should be determined.

The DMFT index is the sum of decayed (D), missing due to caries (M) and filled (F) teeth. Its values range from 0 to 32 and they are measures of oral exposition to cariogenic agents, including bacteria. The number of tooth lesions (D) is the first component of the DMFT index and its values can range between 0 and 32 (similarly to the index values).

To calculate the PI value, a tooth is divided into 4 areas (mesial, distal, lingual and buccal). Each area is assessed for the presence of dental and gingival plaques. The index value is calculated as follows:

$${\text{PI }} = \, \left( {\text{the sum of tooth areas with dental plaque}} \right)/\left( {\text{the number of all tested areas}} \right) \, \times {1}00\%$$

To test the BOP index, a tooth is divided into 4 areas (mesial, distal, lingual and buccal). The BOP presence is assessed for each area. The index value is calculated as follows:

$${\text{BOP }} = \left( {\text{the sum of areas regarding teeth bleeding during the test}} \right)/\left( {\text{the number of all tested areas}} \right) \, \times {1}00\%$$

PPD is assessed on the buccal and palatine surfaces at three sites: mesial, medial and distal.

### Collection, transport and storage of biological materials intended for oral microbiota testing

The biological materials: unstimulated saliva, tongue ridge swab, supra- and subgingival plaques, hard palate swab, palatine tonsil swab and oropharynx swab were collected according to the NIH Human Microbiome Project^16^ protocol with our modifications. Contrary to these researchers, the clinical material from the palatine tonsils was collected at the end of the examination before the posterior pharynx swab collection. The collected biological materials were secured in saline, was placed in a ziplock bag, and placed in portable freezer (BX30, Yeticool, Poland) at − 20 °C. Delivered to the laboratory in about 1 h to the Institute of Molecular Medical Microbiology, Microbiology Department, Jagiellonian University Medical College in Cracow. The samples were then frozen to reach − 80 °C (ULF500 − 86 °C freezer, Infrico medcare, Spain) and stored until further analyses (Table 1). The NIH Human Microbiome Project team^16^ secured the collected materials in the MoBio buffer and placed them in a small ice-filled cooler for transport without defining a precise transport temperature. The material was delivered to the laboratory within about 4 h.

**Table 1:** Protocol for collecting clinical materials from the oral cavity—own study based on the literature^16^.

Mouth area	Required materials	Material collection method	Material protection and transport	Storage of materials pending analysis
Saliva	Sterile 50 ml conical Falcon tube (Labsolute, Germany) Ziplock bag with a capacity of 1 L (Ziploc, USA)	The patient collected unstimulated mixed saliva in the mouth for about 1 min, then spit it out into a test tube. The process was repeated until 2–5 ml of saliva was collected	The capped tube was placed in a ziplock bag, and placed in portable refrigerator (BX30, Yeticool, Poland) at -20 °C. Delivered to the laboratory in about 1 h	Frozen at -80 °C in low temperature freezer (Infrico medcare, Spain) until analysis
Tongue ridge	Sterile flocked swab for sampling (FLOQSwabs, Copan, Italia) Sterile falcon tube with capacity 15 ml (Labsolute, Germany) containing 0.5 ml of sterile saline solution (Symphar, Poland) Ziplock bag with a capacity of 1 L (Ziploc, USA)	We wiped 1 cm^2^ of the middle of the tongue for 10 s, immediately after collection we placed the swab in the tube, pressing it against the tube wall several times for about 20 s to ensure the transfer of bacteria from the swab to the solution	The capped tube was placed in a ziplock bag, and placed in portable refrigerator (BX30, Yeticool, Poland) at − 20 °C. Delivered to the laboratory in about 1 h	Frozen at -80 °C in low temperature freezer (Infrico medcare, Spain) until analysis
Hard palate	Sterile flocked swab for sampling (FLOQSwabs, Copan, Italia) Sterile falcon tube with capacity 15 ml (Labsolute, Germany) containing 0.5 ml of sterile saline solution (Symphar, Poland) Ziplock bag with a capacity of 1 L (Ziploc, USA)	We wiped the entire hard palate for 10 s, placed the swab in the buffer immediately after collection, pressing it against the tube wall several times for 20 s to ensure transfer of bacteria from the swab to the solution	The capped tube was placed in a ziplock bag, and placed in portable refrigerator (BX30, Yeticool, Poland) at − 20 °C. Delivered to the laboratory in about 1 h	Frozen at -80 °C in low temperature freezer (Infrico medcare, Spain) until analysis
Supragingival plaque	Sterile curettes Gracey (MiniGracey 1/2, 11/12, 13/14, LMErgoMax, Finland) Sterile micro tube with capacity 2 ml (Eppendorf, Germany) containing 0.5 ml of sterile saline solution (Symphar, Poland) Silicone air blower (Matin, South Korea) Ziplock bag with a capacity of 1 L (Ziploc, USA)	Samples were taken from 6 teeth (from tooth tissues, not from fillings): 2 molars (first in upper right quadrant and first in lower left quadrant) 2 premolars (first in left upper quadrant and first in lower right quadrant) 2 incisors (they are central in the upper left quadrant and are central in the lower right quadrant) The teeth were isolated with lignin rolls and dried with a gentle stream of air from an air blower. Using the Gracey curette, the entire supragingival plaque was removed from the mesial surface of the tooth. The tip of the curette was immersed in the saline solution for 4–5 s and wiped against the inside edge of the tube. Supragingival plaque samples from six teeth were collected in one tube	The capped tube was placed in a ziplock bag, and placed in portable refrigerator (BX30, Yeticool, Poland) at -20 °C. Delivered to the laboratory in about 1 h	Frozen at -80 °C in low temperature freezer (Infrico medcare, Spain) until analysis
Subgingival plaque	Sterile curettes Gracey (MiniGracey 1/2, 11/12, 13/14, LMErgoMax, Finland) Sterile micro tube with capacity 2 ml (Eppendorf, Germany) containing 0.5 ml of sterile saline solution (Symphar, Poland) Ziplock bag with a capacity of 1 L (Ziploc, USA)	The tooth was isolated with lignin rolls, the surrounding area was dried and the remaining plaque was removed. The mesiobuccal surfaces of six selected teeth were sampled using a Gracey curette. The tip of the curette was immersed in the saline solution for 4–5 s and wiped against the inside edge of the tube. Subgingival plaque samples from six teeth were collected in one tube	The capped tube was placed in a ziplock bag, and placed in portable refrigerator (BX30, Yeticool, Poland) at -20 °C. Delivered to the laboratory in about 1 h	Frozen at -80 °C in low temperature freezer (Infrico medcare, Spain) until analysis
Palatine tonsils	Sterile flocked swab for sampling (FLOQSwabs, Copan, Italia) Sterile falcon tube with capacity 15 ml (Labsolute, Germany) containing 0.5 ml of sterile saline solution (Symphar, Poland) Ziplock bag with a capacity of 1 L (Ziploc, USA)	We rubbed the left and right tonsils for 5 s focusing on the indentations, immediately after collection we placed the swab in the buffer, pressing it against the tube wall several times for 20 s to ensure the transfer of bacteria from the swab to the solution	The capped tube was placed in a ziplock bag, and placed in portable refrigerator (BX30, Yeticool, Poland) at -20 °C. Delivered to the laboratory in about 1 h	Frozen at -80 °C in low temperature freezer (Infrico medcare, Spain) until analysis
Oropharynx	- Sterile flocked swab for sampling (FLOQSwabs, Copan, Italia) Sterile falcon tube with capacity 15 ml (Labsolute, Germany) containing 0.5 ml of sterile saline solution (Symphar, Poland) Wooden tongue depressor 150 mm (Zarys, Poland) Ziplock bag with a capacity of 1 L (Ziploc, USA)	We held the tongue with a wooden depressor, we swabbed the back of the throat for about 5 s, immediately after collection we placed the swab in the buffer, pressing it several times against the tube wall for about 20 s to ensure the transfer of bacteria from the swab to the solution	The capped tube was placed in a ziplock bag, and placed in portable refrigerator (BX30, Yeticool, Poland) at − 20 °C. Delivered to the laboratory in about 1 h	Frozen at − 80 °C in low temperature freezer (Infrico medcare, Spain) until analysis

A precise list of instruments, tube types and activities to perform for proper collection of clinical materials during the intraoral examination, developed by our team, is presented in Table 1. The dental exam and procedures of material collection, its securing and transport supervision were performed by standardized group of dentists.

### Assessment of factors interfering with the NGS results

**Figure 4:**
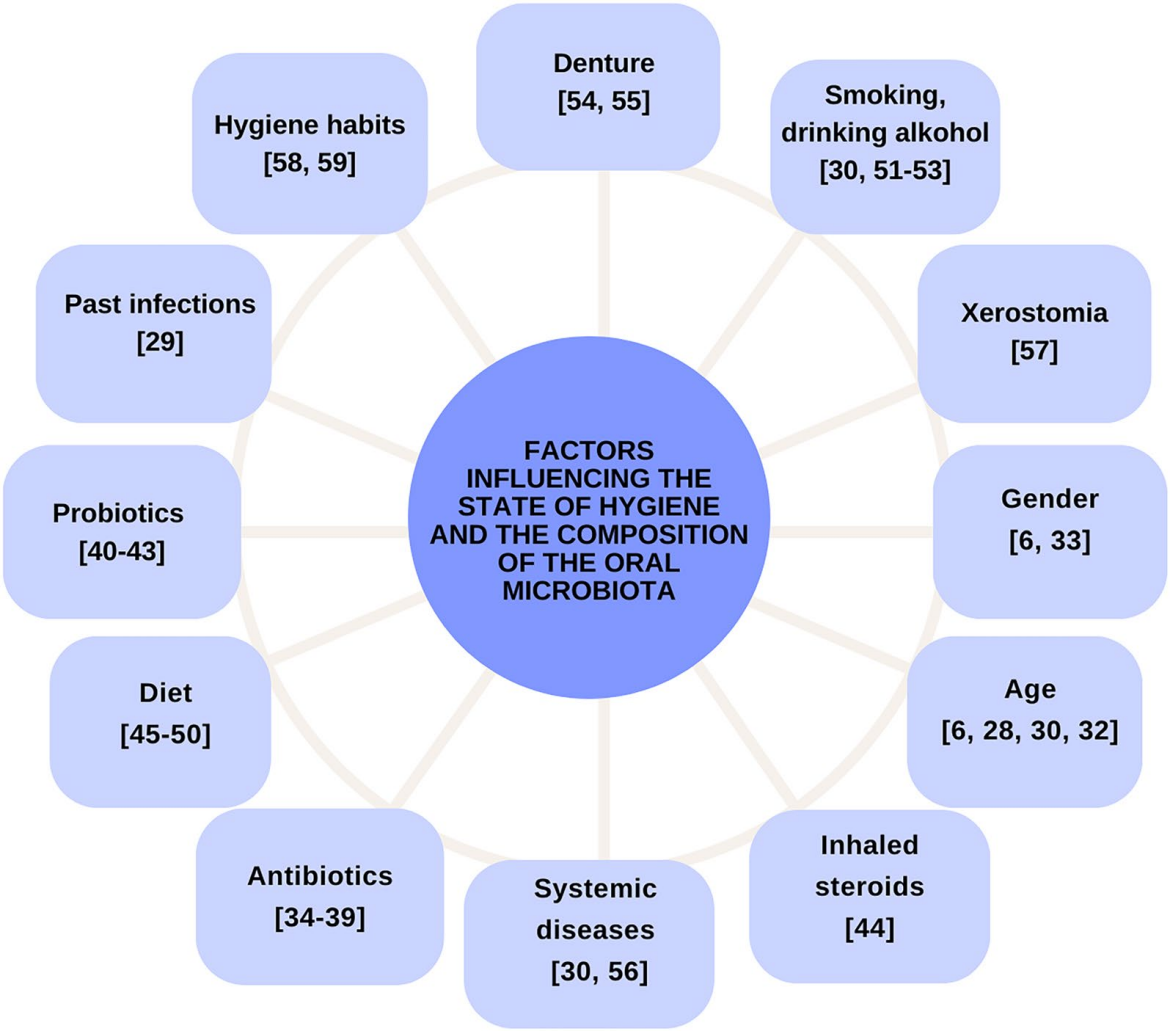
Factors potentially disturbing the results of metagenomic analysis.

In order to standardise the testing conditions and to conduct a reliable analysis, the largest possible number of interfering factors should be considered, e.g. by applying the relevant inclusion and exclusion criteria (Fig. 4).

In our study, the inclusion criteria were: at least 6 teeth present, no oral hygiene activities and fasting for 12 h prior to sample collection, no usage of the denture for at least 12 h prior to the exam, and no consumption of probiotics or antibiotics for at least 3 months prior to the examination. When any of these criteria was not met, the patient was excluded from the study^17^.

### Validation of protocols of clinical material collection for the NGS

The protocols proposed by our team were validated on selected patients as part of the National Center for Research and Development CRACoV-HHS project (Model of multi-specialist hospital and non-hospital care for patients with SARS-CoV-2 infection) through the initiative “Support for specialist hospitals in fighting the spread of SARS-CoV-2 infection and in treating COVID-19” (the contract number: SZPITALE-JEDNOIMIENNE/18/2020). The research was implemented by a consortium of the University Hospital in Cracow and the Jagiellonian University Medical College.

The study involved 60 patients (36 males and 24 females), aged 28–87, assigned to: group I—COVID-19 convalescents who received antibiotics during hospitalisation (n = 17), group II—COVID-19 convalescents without the antibiotic therapy (n = 23), group III—non-COVID-19 healthy individuals (n = 20). The study groups in terms of the age were equivalent (*p* = 0.580). Biological material was collected immediately on the day of the patient's discharge from the hospital, when he was considered, convalescent based on the negative PCR test result for SARS-CoV-2.

### Next generation sequencing methodology

Bacterial DNA was isolated from each clinical sample. The next step was amplification of the bacterial DNA by PCR (the V3–V4 region of the 16S rRNA subunit). The obtained amplicons were used to develop a genomic library in the following stages: purification of PCR products, indexing of the samples and re-purification. Next, the samples were fluorometrically quantified, and the genomic library was pooled for the NGS in the MiSeq platform (Illumina, San Diego, California, United States). The stages of the genomic library development for sequencing are presented in Fig. 1 and have been described previously^17^.

### Bioinformatic analysis and statistical analysis

The statistical analysis of dental indices was carried out using the IBM SPSS Statistics 28. Continuous variables are presented as means ± standard deviations. The study groups were tested by the one-way analysis of variance (ANOVA) with Welch correction, if required, along with the GT2 Hochberg or Games-Howell post-hoc test. The statistical significance was defined as *p* < 0.05 for all the tests. The biostatistical analysis scheme for the post-sequencing operational taxonomic units (OTUs) was described previously^17^.

### Ethics approval and consent to participate

Informed consent was obtained from all participants involved in the study. This research has been approved by the Jagiellonian University Ethical Committee (no. 1072.6120.333.2020 of December 7, 2020). Written informed consent was obtained from all participants, and all methods were carried out in accordance with relevant guidelines and regulations. The study was performed in accordance with the Declaration of Helsinki.

## Results

Selection of the relevant inclusion and exclusion criteria should be considered at the study designing stage to eliminate the largest possible number of interfering factors which can affect the final results of the research. During the dental examination, the order of performed activities was of the utmost importance. Due to its non-invasive nature and no risk of bacterial transfer between the oral habitats, the first assessed parameters were the DMF-T and the tongue coating indices. When the clinical materials from all oral habitats (except the tongue and the tonsils) were secured, the BOP, PI and PPD indices were assessed. Their testing may lead to gingival bleeding and contamination of the tested areas, so it is performed after clinical materials have been collected. The samples from the tonsils and the oropharynx were collected at the end of the exam, which prevented contamination of the other parts of the oral cavity. This might happen as a result of a natural pharyngeal reflex during stimulation of the distal section of the oropharynx.

### Results based on validation of the tested protocols

**Figure 5:**
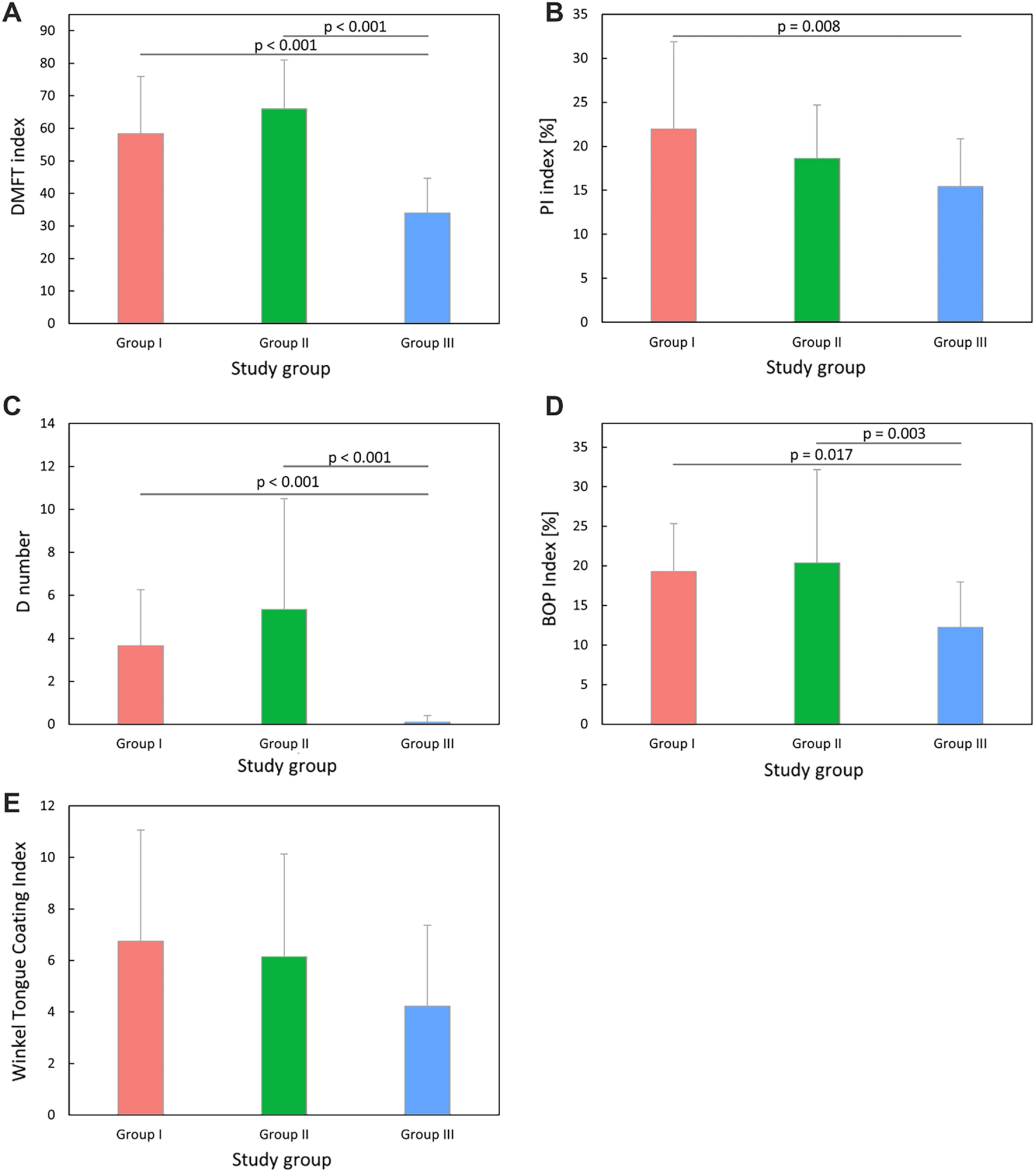
The results of one-way ANOVA for seed dental indices between the study groups.

Statistically significant differences were observed for the dental indices in all study groups: D (*p* < 0.001), DMFT (*p* < 0.001), PI (*p* = 0.011) and BOP (*p* = 0.008). Groups I and II demonstrated statistically significantly higher indices compared to Group III in terms of the DMFT (*p* < 0.001 and *p* < 0.001, respectively), the D number (*p* < 0.001 and *p* < 0.001, respectively) and the BOP (*p* < 0.001 and *p* < 0.001, respectively). In the case of PI, only Groups I and III were statistically significantly different (*p* = 0.008). The PI value was the highest in Group I. The tongue coating index did not show statistical significance between the study groups (Fig. 5).

### Alpha and beta diversities for the selected clinical materials

**Figure 6:**
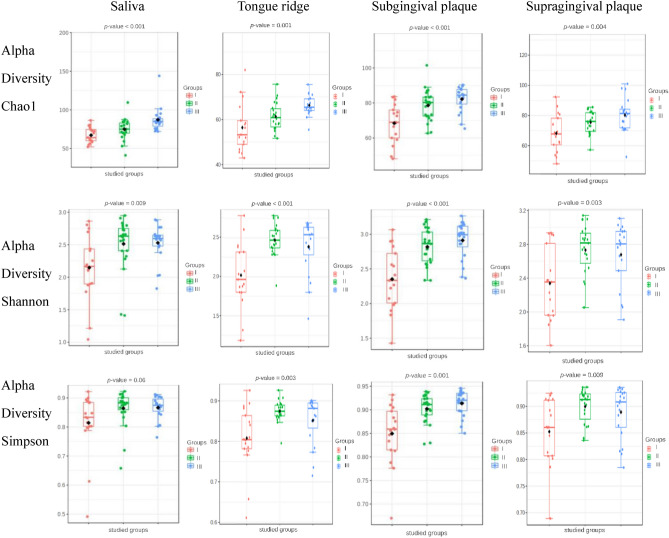
Alpha diversity box plots in the studied groups for selected materials. Legende: The type of the studied groups is shown on the right; centerline, median; box limits, upper and lower quartiles; circle or square symbol, mean; error bars, 95% CI.

Before a detailed analysis of the microbiota of selected oral habitats is conducted, assessment of the alpha and beta diversities is worth consideration as they provide a comprehensive knowledge about microorganisms in a particular environment e.g. the number and diversity of microorganisms in a single sample or habitat (alpha diversity) or the compositional heterogeneity of microorganisms between the samples or habitats (beta diversity). In our study, it was observed (based on the alpha diversity analysis) (Fig. 6) that for each habitat (saliva, tongue, supra- and subgingival plaques), both the numbers of bacteria (expressed by the Chao1 index) and their diversities (expressed by the Shannon and Simpson indices) were statistically lower in Group I than in groups I and II (Fig. 6). An exception was the diversity of microorganisms (only expressed be the Simpson index) in saliva which showed statistical trend towards differences between the study groups (*p* = 0.06). In turn, in group II, the number and diversity of microbiota were similar to those in group III (control) with respect to the tested materials. The only statistically significant differences between these groups were observed in terms of the number of microbiota expressed by the Chao1 index in saliva samples (*p* = 0.005) and tongue swabs (*p* < 0.001) (Table 2).

**Table 2:** Alpha and beta diversity in the studied groups for studied clinical materials.

Materials	Diversity	Index	I group versus II group (*p*-value)	II group versus III group (*p*-value)	I group versus III group (*p*-value)
Saliva	Alpha diversity	Chao1	0.046	0.005	< 0.001
		Shannon	0.020	0.867	0.011
		Simpson	0.100	0.880	0.073
	Beta diversity	Bray–Curtis	0.005	0.091	0.005
		Jaccard	0.002	0.082	0.002
		Jensen–Shannon	0.001	0.103	0.004
Tongue ridge	Alpha diversity	Chao1	0.074	< 0.001	0.001
		Shannon	0.001	0.370	0.007
		Simpson	0.004	0.130	0.073
	Beta diversity	Bray–Curtis	0.001	0.186	0.001
		Jaccard	0.001	0.179	0.001
		Jensen–Shannon	0.001	0.163	0.001
Subgingival plaque	Alpha diversity	Chao1	0.008	0.185	< 0.001
		Shannon	< 0.001	0.257	< 0.001
		Simpson	0.005	0.189	0.001
	Beta diversity	Bray–Curtis	0.001	0.342	0.001
		Jaccard	0.001	0.248	0.001
		Jensen–Shannon	0.001	0.552	0.001
Supragingival plaque	Alpha diversity	Chao1	0.038	0.121	0.010
		Simpson	0.003	0.625	0.013
		Simpson	0.009	0.421	0.054
	Beta diversity	Bray–Curtis	0.005	0.005	0.001
		Jaccard	0.005	0.007	0.001
		Jensen–Shannon	0.007	0.006	0.001

Group I- patients after COVID-19 disease, subjected to antibiotic therapy during treatment; Group II—patients after COVID-19 disease, not subjected to antibiotic therapy during treatment; Group III—healthy patients who have not been infected with the SARS-CoV-2 virus, and have not been treated with antibiotics (control group); *p* < 0.05 the significance level.

**Figure 7:**
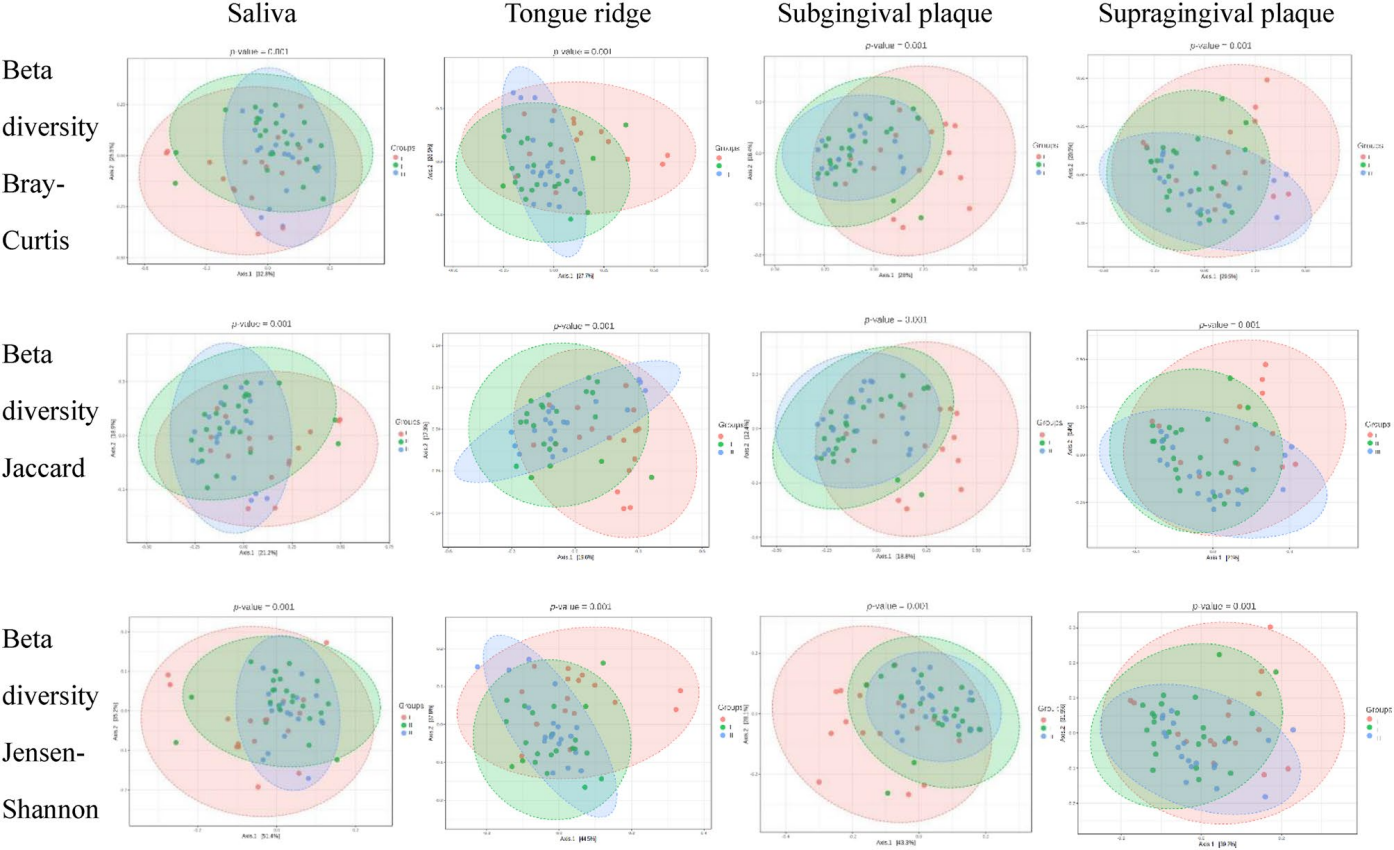
Beta diversity estimated by selected indexes in the studied groups for studied clinical materials.

Beta diversity, as expressed by the Bray–Curtis, Jaccard, and Jensen Shannon coefficients, differed for group I compared to groups II and III in all tested materials, similarly as alpha diversity. Furthermore, statistically significant differences in the diversity of the microbiota were observed between groups II and III only in materials obtained from the supragingival plaque (*p* = 0.005, *p* = 0.007, and *p* = 0.006 for the Bray–Curtis, Jaccard, and Jensen–Shannon coefficients, respectively) (Fig. 7).

## Discussion

### Patient preparation for the dental exam and collection of clinical materials

In our study, the patients fasted before the exam and they did not perform any oral hygiene activities (such as teeth brushing, using oral rinse or cleaning interdental spaces) for at least 12 h and did not use any dentures to eliminate local agents which might affect the microbiota composition. These activities are often neglected by other researchers^16,18–22^. In the study conducted by Ren et al*.*^23^, the participants used sterile water for double mouth rinsing before the collection of material from the tongue^23^. The patients assessed by Ma et al*.*^22^ rinsed their throats with clean water before the collection of posterior pharynx swabs as well as the swabs from the lateral walls and crypts of the tonsils. In our research, contrary to other studies, the material was collected by a trained dentist to maintain the highest standards. A limited number of dentists will ensure that collection-related errors are avoided/limited, which will guarantee more homogenous groups of materials.

### Dental examination

An additional component of the dental exam ensuring a wide understanding of a patient’s oral and dental health was the assessment of the DMFT (Decayed, Missing and Filled Teeth) index which is recommended by the WHO and used worldwide as the most important indicator of tooth decay prevalence. The DMFT value is affected by many factors appearing during a patient’s lifetime. It reflects the patient’s oral health and influences the microbiota composition. Therefore, to obtain a complete knowledge, we also assessed other indices which describe the oral cavity depending on the bacterial dental and gingival plaques and plaque-induced diseases. The indices were as follows: PI, BOP and PPD. In addition, we assessed the WCTI values. Our study was the first research to correlate the clinical material collected from various oral habitats with the dental indices.

### Collection of biological material from various oral areas

In our previous study^17^, we draw attention to the types of biological material collected by other researchers and their usefulness for oral microbiota analyses. Biological materials, such as oral rinse samples^18^, saliva^19–24^ or oral swabs^23^ and sometimes only posterior pharynx swabs were collected^20,25^. This kind of clinical material selection and an unclear description of the collection method may cause errors in the interpretation of results and their comparison with other research studies.

### The order of material collection

In our study, we were particularly focused on the order of exam activities and on securing biological material for metagenomic testing.

The first assessed parameters were the DMFT and WTCI values as their testing procedures are non-invasive, do not contaminate the area being assessed due to contamination of materials between the habitats and do not lead to bleeding which would affect the other habitats. Next, we collected clinical material from all other habitats except the palatine tonsils and posterior pharynx. Then, we assessed the PI, BOP and PPD indices. In the case of periodontitis, which is manifested by enhanced gingival bleeding, assessment of these indices may result in bleeding and prevent collection of non-contaminated biological material. Not all authors took this into consideration, e.g. Caselli et al*.*^1^ secured the clinical material following assessment of the BOP index.

We collected samples from the tonsils and the oropharynx at the end of the dental examination. Proper collection of this material was the most difficult procedure in practice. Following collection of the material from the tonsils (which caused discomfort), the patients reluctantly agreed to have the pharynx swab. We collected both materials at the end of the dental exam because while securing them, other oral habitats may become contaminated due to the pharyngeal reflex. The authors of the protocol of the NIH Human Microbiome Project^1^ also emphasise the importance of collecting the posterior pharynx swab at the end of the exam but they do not mention the palatine tonsil swab. In our study, we also observed pharyngeal reflexes in the study participants during swab collection from the tonsils so we suggest this procedure should be performed at the end of the exam prior to pharyngeal material collection. Another order of the exam activities may result in material contamination and erroneous results when such a sensitive method as the NGS is applied.

### Transport and storage of biological material

Properly selected temperature conditions for sample transport should possibly best reflect a patient’s clinical status and ensure optimal conditions for the survival of microorganism and their genetic material without multiplication of its components. To achieve this, samples are usually cooled to reach + 2–8 °C^1^. Our experience in oral biological sample preparation shows that the transport of material at even lower temperature (− 20 °C) and its storage at − 80 °C prevented bacterial DNA degradation, which is a key aspect of metagenomic testing. Moreover, in our previous microbiota studies, the storage of various clinical materials (including blood, faeces or intestinal biopsy) at − 80 °C prolonged the DNA isolate stability and did not negatively affect the results of our analyses^26,27^. The material which is secured using this method can be stored for many months without the loss of its value^28,29^.

### Assessment of the interfering factors

Various factors may affect a result of oral microbiota assessment (Fig. 4). A patient’s age or gender cannot be eliminated. The oral microbiota composition changes with age^28,29^. The age-related dental symptoms, e.g. xerostomia, teeth loss, poorer immune system and adverse affects of drugs, have long-term effects on the oral health^6^. In a meta-analysis conducted by the team (authors), it was observed that additionally, COVID-19 disease significantly deepens the differences in the composition of the oral microbiota, especially in older people > 60 years of age compared to the group of patients < 60 years of age. These changes mainly involve a reduction in alpha diversity and an increase in the imbalance of the oral microbiome, which consequently leads to an increase in the level of opportunistic pathogens, including: Enterococcus, Enterobacter, Streptococcus, Veillonella, Prevotella Porphyromonas and Aggregatibacter^30^.

In addition, the incidence of systemic diseases becomes higher with age, which results in a higher risk of poor oral health^31^. Interestingly, based on a review (author et. al.), it was proven that the peri-implant microbiota is different from the periodontal microbiota. These differences are manifested (in the case of pre-implant) by lower bacterial diversity, but at the same time by a larger population of peridontopathogenic bacteria and bacteria belonging to, among others, to the classes: Bacteroidia, Spirochaetes, Synergistia, Clostridia and Deltaproteobacteria. In turn, healthy peri-implant sites are dominated by bacteria belonging to the Actinomycetia class. Regardless of health status, the microbiota becomes more complex as inflammation progresses (from mucositis to peri-implantitis)^32^.

The age-related exacerbation of periodontitis has been observed^33^. According to Skorupka et al*.*^6^, the population aged over 65 years demonstrates a much higher negligence regarding oral hygiene and care^6^. They also observed that women paid much more attention to oral hygiene, and they had many more own teeth than men^6^, which resulted in higher incidence rates and higher severity of periodontal diseases in men compared to women^34^. Men demonstrate worse periodontal parameters: the plaque index, BOP and PPD are significantly higher in men^34^.

Among the factors which can be eliminated, it has been proved that there are factors greatly affecting the oral health and microbiota. One of the best-known interfering factors in the microbiota composition is the antibiotic therapy^35^. It may induce reduction of the diversity of oral microbiota and change microbial functions^36–39^. While numerous reports on the impact of antibiotic therapy on the microbiota of the gastrointestinal tract, mainly the intestines, are available^40^, its impact on the oral microbiota remains poorly understood^35^.

Contrary to few studies assessing the effects of microbiota on the oral health, the impact of probiotics on oral microorganisms is better documented. The metaanalysis of 554 patients shows that oral probiotics did not lead to significant improvement in the GI, PI, and BOP values in the patients with plaque-induced gingivitis and microbiota^41^. However, some studies prove that the use of selected oral probiotics might promote additional clinical and immunological benefits while treating generalized gingivitis^42,43^.

According to the scientific reports, another factor affecting the oral health is the use of inhaled steroids for asthma treatment. They demonstrate a strong anti-inflammatory effect. Patients with asthma are at the fivefold higher risk of periodontitis than healthy individuals^44^. In 83% of the patients taking asthma medications, the chance of periodontitis diagnosis was smaller than in individuals who do not receive medications regularly^44^.

A diet is one of the factors affecting the diversity and interactions of the oral microbiota^45^. It has been proved that the diet is a factor which modifies e.g. the course of tooth decay, teeth erosions or periodontal diseases^46^. Indeed, the high-sugar diet adversely influences the oral microbiota balance^45^ and increases the risk of tooth decay^47^. The abundance and diversity of oral microorganisms were significantly lower in the individuals with a higher sugar intake^48,49^. Anderson AC et al*.*^50^ observed an increased number of *Streptococcus* species and decreased numbers of *Proteobacteria*, *Pasteurellaceae*, *Bacteroidia* and *Porphyromonas* species in individuals on the high-sugar diet^50^.

Tobacco and alcohol may disturb the oral and pharyngeal microbiota composition and lead to a chronic inflammation^51^. The oral mucosal microbiome participates in production of genotoxic acetaldehyde through alcohol oxidation and caused DNA damage by producing DNA adducts in the oral mucosal cells^52^. Chronic smoking changes the oral microbiota to produce higher amounts of acetaldehyde from alcohol^53^. The species greatly associated with a higher production of acetaldehyde were *Streptococcus salivarius*, *Streptococcus* sp., *Corynebacterium* sp., *Stomatococcus* sp. and yeast^53^. The oral health is highly affected by poor hygiene and the use of dentures. The polymicrobial biofilm may proliferate on the surfaces of dental materials and form a bacterial plaque, which stimulates local inflammations clinically manifested by erythema and hyperplasia^54^. Prosthetic stomatitis shows a clinically significant relation to tooth decay, periodontitis, median rhomboid glossitis, cheilitis as well as aspiration pneumonia and its mortality^55^. Therefore, we recommend against using dentures for 12 h prior to the dental exam.

Systemic diseases such as diabetes, rheumatoid arthritis (RA) and systemic lupus erythematosus (SLA) increase the risk of periodontal diseases^56^. They exacerbate periodontitis symptoms and increase its risk or severity^56^. Diabetes-related xerostomia causes oral discomfort and is associated with swallowing disorders, malnutrition and exacerbated oral symptoms^57^, which affects saliva collection. Therefore, we suggest creating a separate group of patients with selected systemic diseases during patients’ enrolment in the study.

Additional hygienic activities improve the oral health. The use of sugar-free chewing gum as an addition to teeth brushing ensures a small but significant reduction of the dental plaque^58^. Moreover, the use of antiseptic oral sprays containing 0.2% chlorhexidine leads to reduction of the dental plaque (PI) and gingival (GI) indices^59^. The use of natural substances such as probiotics, paraprobiotics, ozonated substances, and postbiotics reduce the percentage of pathological bacteria, consequently enabling the restoration of homeostasis of the oral microbiota. In the future, such supportive treatment may be implemented in people with microbiota disorders, including people with periodontal diseases^60,61^.

Depending on the study objectives, selection of the relevant inclusion and exclusion criteria as well as the kinds of material for collection is necessary to ensure the most reliable results of the microbiome analysis. Thus, while applying comprehensive analyses of the alpha and beta diversity, determination of the effects of a specific factor/disease on the number and diversity of microorganisms within the study group as well as a comparison of the results in the aspect of the other study groups are possible. In our study, based on the results obtained, it can be concluded that both COVID-19 disease and the use of antibiotics have an impact on the oral microbiota diversity and composition. Post-COVID-19 patients, especially those who received antibiotics, exhibited significantly lower abundance and diversity of oral microorganisms, as well as a more homogeneous microbiota composition, compared to the healthy study participants. We have also observed that materials collected from the supragingival plaque are particularly sensitive to changes in the microbiota caused by both COVID-19 and antibiotics.

Summing up, due to an increasingly wider application of the metagenomic analyses based on the sequencing findings and the absence of obligatory schedules and procedures for the oral microbiome testing, a detailed determination of the inclusion and exclusion criteria for the study and control groups (to eliminate the interfering factors) as well as a strict observance of the order and methodology of biological material collection from the individual oral habitats are crucial.

## Conclusion

Due to different schedules of clinical material collection applied by various researchers for metagenomic testing, standardisation of the method and a strict observance of the protocols are necessary to avoid technical problems as well as to obtain reliable and reproducible testing results. The order of activities according to the dental exam protocol and the order of activities to secure materials collected from individual oral habitats are particularly important to avoid material contamination which considerable disturbs the microbiome testing results.

Based on our study, it has been shown that the DMFT, D number, PI and BOP indices are good prognostic parameters in the assessment of oral health. The analysis of the beta diversity showed that in all study groups, the supragingival plaque was the most sensitive material to changes in the composition of microorganisms.

At the stage of study designing, it is necessary to consider the highest possible number of factors which may disturb the oral health as they will lead to erroneous results and conclusions.

An appropriate method of collecting clinical samples for NGS testing is the basis for obtaining reliable material reflecting the actual condition of the oral cavity. Therefore, the type, sequence and method of collecting, securing and transporting samples from individual areas of the oral cavity, as well as the tools used for this purpose, are important. Moreover, for the full interpretation of the results, indicators describing the clinical condition of the oral cavity at the time of material collection are also important, as it may be a local factor modifying the microbiota.

Our article was inspired by the publications of other researchers, which showed that there is no such standard. Microbiological samples were collected in various ways, but all were defined under the general name of oral samples. Meanwhile, we know that there are different surfaces and niches in the oral cavity, inhabited by different microbiota. Taking into account the above arguments, we decided that sharing our experiences may help other research teams in improving methods of obtaining and examining microbiological samples from the oral cavity.

### Supplementary Information

Supplementary Information.

## Data Availability

Supplemental data for this article can be accessed online at https://www.ncbi.nlm.nih.gov/bioproject/PRJNA997828, Accession PRJNA997828.

## Abbreviations

NGS: Next generation sequencing
DMFT: Decayed, missing and filled teeth index
D number: The number of tooth lesions D (decayed)
PI index: The plaque index simplified
BOP index: Bleeding on probing index
WTCI: Winkel tongue coating index
PPD: Periodontal probing depth
