# Supplementary material for: Standardization of the protocol for oral cavity examination and collecting of the biological samples for microbiome research using the next-generation sequencing (NGS): own experience with the COVID-19 patients
Source: Sci Rep. 2024 Feb 14;14:3717. doi: 10.1038/s41598-024-53992-3 (PMC10867075; doi:10.1038/s41598-024-53992-3)
Supplement: Supplementary file 1 — Supplementary Information. [file 41598_2024_53992_MOESM1_ESM.pdf]

EXAMINATION CARD / SURVEY CARD OF DENTAL EXAMINATION

A. Information about the patient and the applied therapy

Name and surname			Examination date
Date of birth			Age
Gender			Date of admission to the hospital
☐ Male ☐Female			
Patient card number (according to the marking of the examination at the place of hospitalization)			Patient's card number (as marked by own research)
Reason for hospitalization			
Antibiotic use in the last 3 months			Probiotic use in the last 3 months
☐ Yes ☐ No			☐ Yes ☐ No
The name of the antibiotic used			The name of the probiotic used
Comorbidities/chronic diseases			Drugs taken chronically
Pharmacology prescribed during hospitalization			
Medicine name	Dose of medicine	Application time	Route of administration

B. Clinical examination (intraoral)

1. DMFT index (Decayed Missing Filled Teeth index)

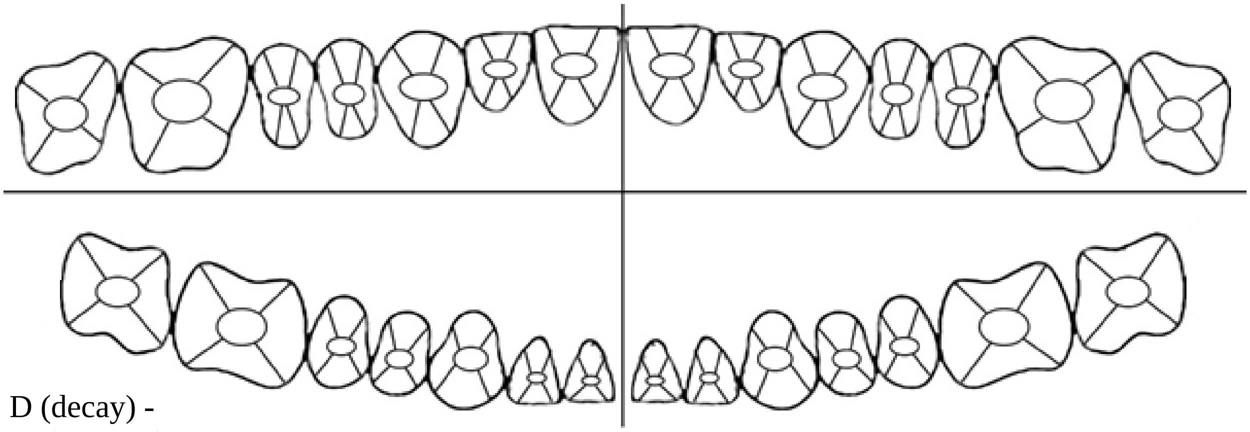

D (decay) -
M (missing) -
F (filled) -

DMFT= D+M+F+T=

*The extracted tooth should be crossed out on the diagram

2. Winkel Tongue Coating Index (WTCI index)

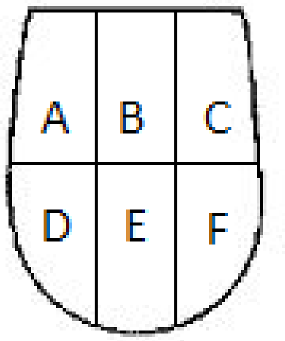

Tongue type:
- ☐ - geographical
- ☐ - cerebral
- ☐ - black
- ☐ - encased
- ☐ - atrophic
- ☐ - none of the above

A-<br>B-<br>C-<br>D-<br>E-<br>F-	Legend:<br>0 - not coated<br>1 - thin coated<br>2 - thick coated

WTCI= A+B+C+D+E+F=

3. BOP index (Bleeding on Probing)

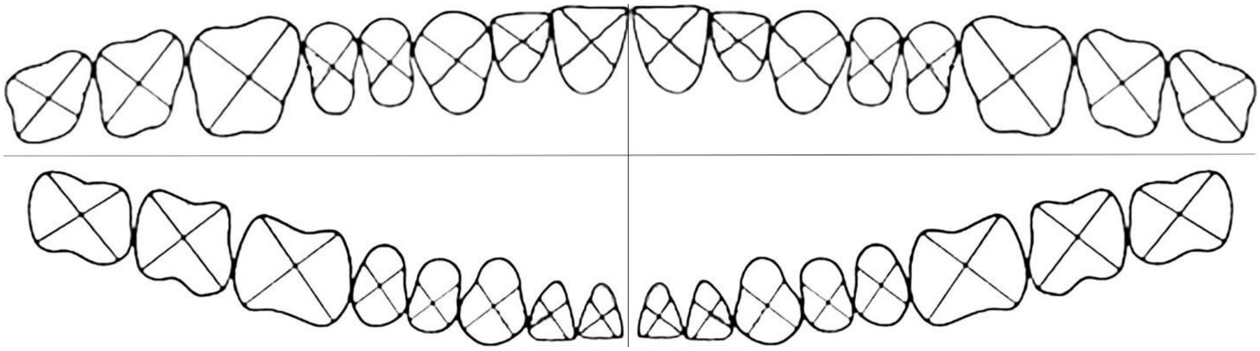

BOP = — x 100% =

*tick ,,+" the appropriate areas

4. PI index (Plaque Index simplified)

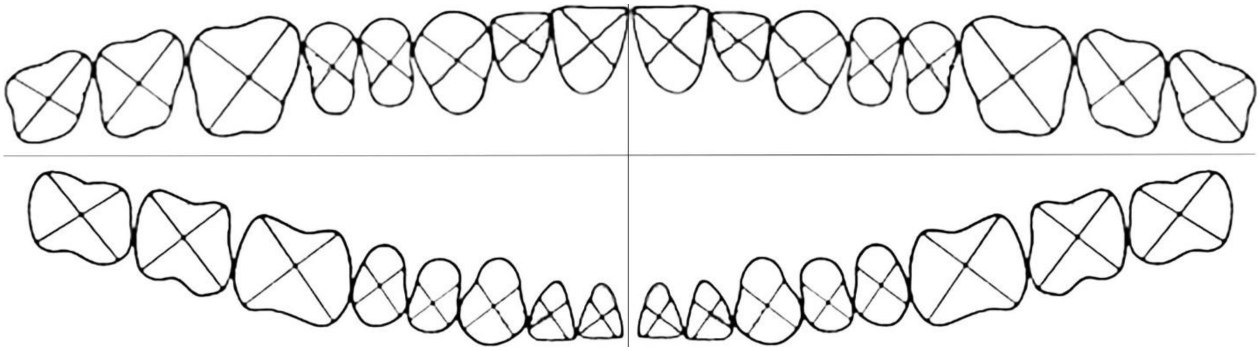

PI = — x 100% =

*tick ,,+" the appropriate areas

5. PPD (Periodontal Probing Depth)

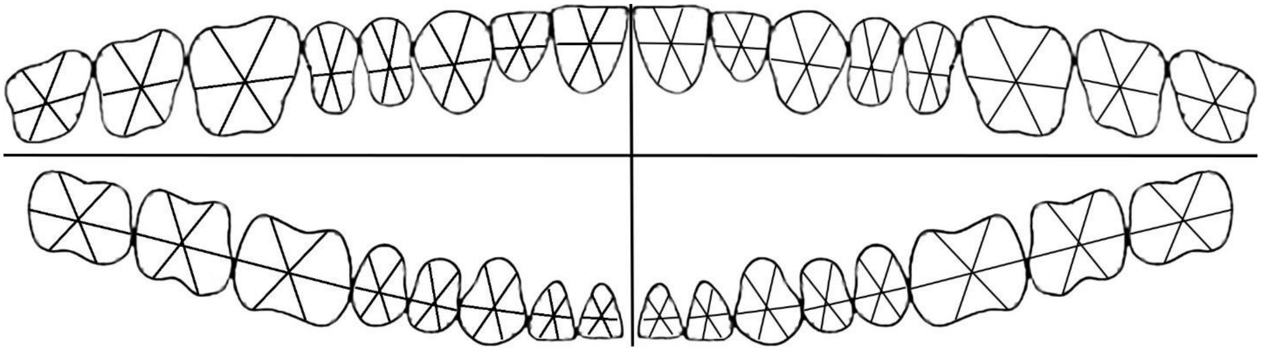

*enter the tested value in the appropriate areas

6. The use of dentures

Yes ☐

No ☐
